# Differences in Clinical Presentations of Omicron Infections with the Lineages BA.2 and BA.5 in Mecklenburg-Western Pomerania, Germany, between April and July 2022

**DOI:** 10.3390/v14092033

**Published:** 2022-09-13

**Authors:** Katja Verena Goller, Juliane Moritz, Janine Ziemann, Christian Kohler, Karsten Becker, Nils-Olaf Hübner, the CoMV-Gen Study Group

**Affiliations:** 1 Central Unit for Infection Control and Prevention, University Medicine Greifswald, 17475 Greifswald, Germany; 2 Friedrich-Loeffler-Institute of Medical Microbiology, University Medicine Greifswald, 17475 Greifswald, Germany; 3 Institute for Hygiene and Environmental Medicine, University Medicine Greifswald, 17475 Greifswald, Germany

**Keywords:** SARS-CoV-2, Omicron, BA.2, BA.5, symptoms, Germany

## Abstract

Knowledge on differences in the severity and symptoms of infections with the SARS-CoV-2 Omicron variants BA.2 (Pango lineage B.1.529.2) and BA.5 (Pango lineage B.1.529.5) is still scarce. We investigated epidemiological data available from the public health authorities in Mecklenburg-Western Pomerania, Northeast Germany, between April and July 2022 retrospectively. Comparative analyses revealed significant differences between recorded symptoms of BA.2 and BA.5 infected individuals and found strong correlations of associations between symptoms. In particular, the symptoms ‘chills or sweating’, ‘freeze’ and ‘runny nose’ were more frequently reported in BA.2 infections. In contrast, ‘other clinical symptoms’ appeared more frequently in Omicron infections with BA.5. However, the results obtained in this study provide no evidence that BA.5 has a higher pathogenicity or causes a more severe course of infection than BA.2. To our knowledge, this is the first report on clinical differences between the current Omicron variants BA.2 and BA.5 using public health data. Our study highlights the value of timely investigations of data collected by public health authorities to gather detailed information on the clinical presentation of different SARS-CoV-2 subvariants at an early stage.

## 1. Introduction

Starting in December 2021 and ongoing until July 2022, infections with the Severe Acute Respiratory Syndrome Virus 2 (SARS-CoV-2) in Germany have mainly been caused by the Omicron variant (Phylogenetic Assignment of Named Global Outbreak (Pango) lineage: B.1.1.529) and its subvariants BA.1, BA.2, BA.3, BA.4 and BA.5, including their respective sub-lineages [1]. In December 2021, the Omicron subvariant BA.2 was reported for the first time in Germany [1,2]. Since then, it has rapidly replaced the Omicron subvariant BA.1 that had been dominating until then and was the dominant variant by the end of February 2022 [1]. Concurrently, by the end of February 2022, the Omicron variant BA.5, first detected in April 2022 in South-Africa where it was responsible for a fifth wave between April and June 2022 [3,4], appeared in Germany [1]. The BA.5 variant increased rapidly and became the dominating variant in Germany by calendar week 23 (June 2022) [1].

Little is known about differences in the severity of disease between BA.2 and BA.5 infections. However, a study in animals recently suggested that BA.4 and BA.5 may be more pathogenic than BA.2 [5], and there is also evidence that the Omicron variants BA.4 and BA.5 exhibit a higher transmissibility than BA.2 [6].

Interestingly, to the best of our knowledge, differences in clinical presentations between the currently circulating Omicron variants BA.2 and BA.5 have not yet been reported on the basis of public health data. We therefore conducted a comparative analysis between BA.2 and BA.5 lineages using official reporting data from the public health authorities from the German federal state Mecklenburg-Western Pomerania.

## 2. Materials and Methods

In order to investigate differences in symptoms caused by infections with the Omicron lineages of BA.2 and BA.5, including their respective sub-lineages, data available from the public health authorities from the federal state Mecklenburg-Western Pomerania, Germany, were used. For the German federal state of Mecklenburg-Western Pomerania, the CoMV-Gen Study Group (www.comv-gen.de (accessed on 8 August 2022)) is entrusted by the state government with monitoring the circulating SARS-CoV-2 -variants in collaboration with local diagnostic laboratories and public health authorities. On behalf of the state government, the CoMV-Gen project’s mission is conducting the SARS-CoV-2 variant surveillance in Mecklenburg-Western Pomerania, including gathering genetic information from target PCRs as well as from whole genome sequencing analyses in collaboration with local diagnostic laboratories, and connecting these with epidemiological data available from the public health authorities. All confirmed positive cases recorded between 25 April and 13 July 2022 (covering twelve calendar weeks) in the public health surveillance system (Surveillance Outbreak Response Management and Analysis System (SORMAS) [7]) by the public health authorities from Mecklenburg-Western Pomerania were analyzed retrospectively. Data on symptoms were mainly collected by structured self-reporting questionnaires, and to a lesser extent, from interviews with the cases. Data from individuals that were notified as being infected by Omicron were collected. All cases reported as being infected by Omicron BA.2 or BA.5 lineages up to 13 July 2022 were considered for analyses. According to the standardized and cross-sectional German Corona Consensus Dataset (GECCO) [8] for research, symptoms relevant to COVID-19 were filtered out and frequencies were compared. In order to investigate the infection status across the years of age of the positive cases, five age groups in 20-year intervals were defined: 0–19, 20–39, 40–59, 60–79 and 80 and more years of age.

Ethical approval was granted by the ethics committee of the University of Greifswald, Germany (BB 125/21). Only anonymized aggregated data are shown in this manuscript and only symptomatic cases were used to compare symptoms when the exact lineages were assigned and reported in the system either as BA.2 or BA.5.

Statistical analyses were conducted by using SPSS 28.0 (IBM SPSS Statistics for Windows, Version 28.0. Armonk, NY, USA: IBM Corp.). For comparative analyses, Pearson’s chi-square tests were conducted, and the 95% confidence intervals (CI95) were determined A *p*-value of 0.05 was considered statistically significant. In order to analyze correlations between symptoms for both variants, non-parametric Spearman Rho correlation coefficients were determined for symptoms that occurred with a minimum overall frequency of 10% of cases.

## 3. Results

**Figure 1:**
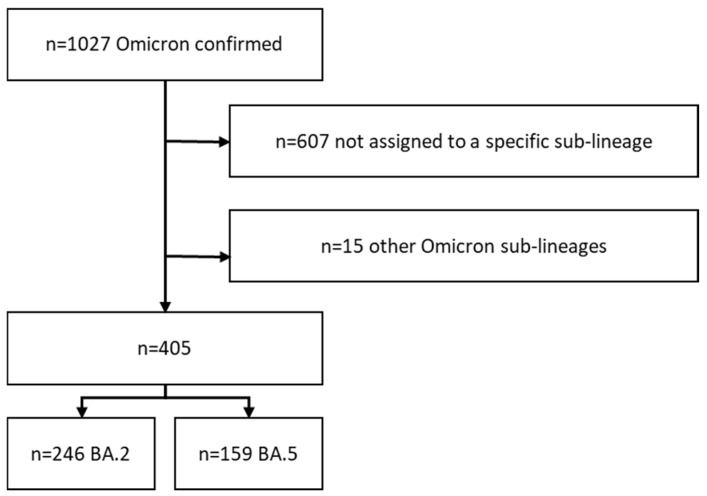
Sample size of the study population. In total, 405 assigned BA.2 and BA.5 cases were included in the analyses.

In total, 1027 cases with confirmed Omicron infections were reported in SORMAS during the study period (Figure 1). Of those, 607 cases were deposited as Omicron-positive in SORMAS but were not assigned to a specific sub-lineage, while 405 cases were characterized as BA.2 (*n* = 246) or BA.5 (*n* = 159), respectively. Fifteen cases belonged to other Omicron lineages including BA.1 (six cases) and BA.4 (nine cases). These 15 cases, as well as the unassigned cases, were excluded from the analyses.

Information on infection status per age group, sex, symptomatic and vaccination status are summarized in Table 1. Statistical analyses revealed no significant differences between the age groups, sex and vaccination status of BA.2 and BA.5 cases (Table 1). Within the BA.2 cases 124 individuals (50.1%), and of the BA.5 cases 69 individuals (43.3%) reported one or more symptoms (Pearson’s chi square statistics: *p* = 0.561). 

A summary of the statistical analyses including CI95 intervals for each symptom and results from Pearson’s chi-square statistics are given in Table 2. No records for ‘acute respiratory distress syndrome’, ‘respiratory insufficiency/assisted ventilation’ and ‘oxygen saturation < 94%’ were given. 

**Figure 2:**
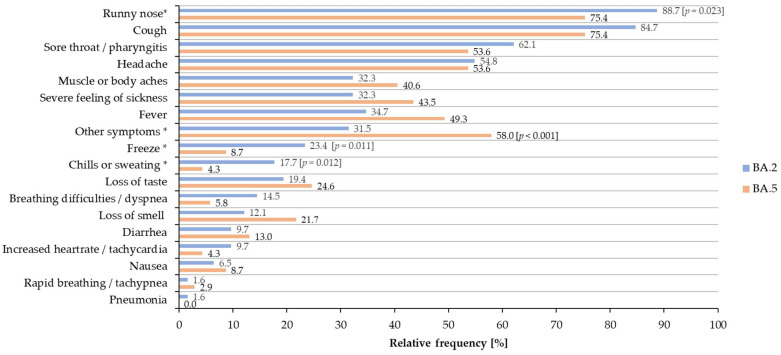
Relative frequency of the most prominent 18 symptoms of infected symptomatic BA.2 and BA.5 cases. * *p* values are given in squared brackets for significant results.

The absolute and relative frequency of the symptoms of cases of our study population either infected with BA.2 or BA.5 are additionally shown in Figure 2. Altogether, 21 specific symptoms (according to the GECCO-criteria [8]) are of relevance for COVID-19 infections and the content of the questionnaire. The most prominent symptoms reported in BA.2 as well as BA.5 cases were respiratory symptoms such as ‘runny nose’, ‘cough’ and ‘pharyngitis’. On the other hand, severe symptoms such as ‘acute respiratory distress syndrome’, ‘oxygen saturation below 94%’ and ‘respiratory insufficiency that needs assisted ventilation’ were not reported for both variants.

However, the Omicron BA.2 and BA.5 lineages also showed marked differences in the frequency of various symptoms, as shown in Table 2 and Figure 2. Significant differences in symptoms ‘chills or sweating’ and ‘freeze’ between BA.2 and BA.5 infections were observed. These symptoms were more frequently notified from individuals infected with BA.2 (‘chills or sweating’: *p* = 0.012; 17.7%, CI95: 11.8%–25.2%; ‘freeze’: *p* = 0.011; 23.4%; CI95: 16.6%–31.4%) than from BA.5 cases (‘chills or sweating’: 4.3%, CI95: 1.2–11.1%; ‘freeze’: 8.7%; CI95: 3.7–17.0%), with 88.7% of BA.2 cases more frequently reporting ‘runny nose’ (*p* = 0.023; CI95: 82.3–93.4%) than BA.5 cases (75.4%; CI95: 64.3–84.3%). In contrast, BA.5 cases significantly more often reported ‘other symptoms’ (*p* < 0.001; 58.0%, CI95: 46.2–69.1%) than BA.2 cases (31.5%, CI95: 23.8–40.0%). ‘Other symptoms’ included a wide range of symptoms such as ‘dullness’, ‘exhaustion, ‘fatigue’, ‘dizziness’ among others that are all not items in the GECCO catalogue. This might indicate a more elaborated set of symptoms reported from BA.5 cases.

Correlations between symptoms for both variants are shown in Table 3.

As shown in Table 3, ‘loss of smell’ was strongly correlated with ’loss of taste’ (Spearman Rho = 0.571, *p* < 0.01); ‘freeze’ was strongly correlated with ‘chills or sweating’ (Spearman Rho = 0.579, *p* < 0.01), as well as with ’muscle or body aches’ (Spearman Rho = 0.328, *p* < 0.01); and ‘headache’ was correlated to ‘runny nose’ associated with ‘cough’ (Spearman Rho = 0.334, *p* < 0.01).

## 4. Discussion

In this study, we conducted a comparative analysis of the clinical presentations between the Omicron BA.2 and BA.5 lineages, including their respective sub-lineages, using official reporting data from the public health authorities of the Germany federal state Mecklenburg-Western Pomerania. To the best of our knowledge, this is the first time that the differences in the clinical presentations between these two current Omicron lineages have been reported using public health data.

Both sub-lineages showed a comparable severity of infection with approximately 50% of cases being symptomatic. Overall, the symptoms ‘runny nose’, ‘cough’, ‘sore throat’ and ‘headache’ were most often reported by symptomatic cases in both variants, while ‘pneumonia’ and other symptoms of a severe course of the disease were rare or not reported. However, the BA.2 and BA.5 lineages showed that differences in the frequency of certain symptoms—e.g., ‘other symptoms’ that are not part of the GECCO list of relevance for COVID-19 infections—were reported more frequently from BA.5 than from BA.2 cases. In contrast, the symptoms ‘chills or sweating’, ‘freeze’ and ‘runny nose’ were more frequently reported in BA.2 cases. This fits well with the literature, with ‘headache’, ‘runny nose’, ‘sore throat’, ‘sneezing’, ‘persistent cough’ and ‘fever’ as the most prominent symptoms reported for COVID-19 infections [9].

Our analysis shows some strong correlations between symptoms. Some of these correlations can be explained by the fact that they are part of the respiratory symptom cluster [10], and some are clearly distinguishable but closely related, such as ‘loss of smell’ and ‘loss of taste’, respectively. Others can be explained by the fact that they are difficult for patients to distinguish, e.g., ‘freeze’ and ‘chills or sweating’, and some can be interpreted as a distinctive clinical course, e.g., ‘chills or sweating’ and ’muscle or body aches’. Other studies most prominently reported SARS-CoV-2 symptoms such as ‘loss of smell’ and ‘loss of taste’, mainly when associated with ‘fever’, and ‘cough’ in association with ‘shortness of breath’ and ‘chest pain’ [10]. However, different SARS-CoV-2 variants are known to exhibit different symptoms and influence the course of infections [2,11,12]. For BA.2, the European Centre for Disease Prevention and Control (ECDC) reports reduced evidence and for BA.5 no evidence for the impact on severity so far [13]. However, different variants and sub-variants have genetically determined differences that have been shown to correlate with different clinical features and outcomes [5,11].

Our study has several limitations that should be considered for the interpretation: First, the estimated differences in symptoms between the BA.2 and BA.5 lineages could be biased due to a low incidence situation at the beginning of a new wave. Second, the database provided by SORMAS is mainly based on self-reports of the patients who were either interviewed by the public health authorities staff or filled in standardized questionnaires on their own. In particular, the recording of symptoms depends on the timing of the query, the patient’s physical condition and their willingness to provide information. As the public health departments and the use of SORMAS is decentralized, the way in which data were collected (either by interview or questionnaire) may have some differences between counties and over time. Although response is mandatory, it is quite possible that, for example, severe cases who were too ill to answer the questions were not fully recorded, as hospital record data can, but which is not systematically used. Since the questioning is one-time after the notification date, it is also conceivable that, depending on the day of the response of the patient, not all symptoms were recorded; later, potential hospitalization during the course of infection may, thus, not have been reported. Another possible systematic error is the partial overlap of symptoms in the symptom categories. For example, the symptom ‘freeze’ resembles the symptom ‘chills’ and, thus, could be reported either in both categories or only in one. This could also explain the strong correlation between both symptoms. Third, only cases about which data on the Omicron lineage were present were included in the study. Therefore, only a small number (less than 5%) of confirmed cases could be included. This could aggravate the fact that, to date, many people use commercial self-testing systems when acquiring respiratory or other typical symptoms for COVID-19 infections and may, in some cases, not report their infection status to the public health authorities. Additionally, sub-lineages in the BA.2 and BA.5 groups were pooled in our analysis, despite the fact that the genetic heterogenicity amongst these sub-lineages steadily increased over the study period.

However, our study has some strengths: The data comprised official reporting data for a geographically distinct region and a manageable time frame. The Omicron lineages were well comparable in terms of age and sex structure, vaccination and frequency of symptomatic cases. Thus, it is not to be expected that external, undetected influences have significantly distorted the results.

In summary, our results provide no evidence that BA.5 has a higher pathogenicity than BA.2. However, approximately 50% of cases developed symptoms which could lead to sick leave. If the incidence is high, this could represent a significant social burden, especially in critical infrastructure and healthcare areas. Differences in symptoms between variants should continue to be closely monitored in order to identify symptomatic patterns early.

## 5. Conclusions

To our knowledge, we firstly report profound differences in symptoms between the Omicron lineages BA.2 and BA.5 based on public health data. Our results show some significant differences between the lineages but provide no evidence that BA.5 leads to a more severe course of disease than BA.2. Our study highlights the opportunities and challenges of analyzing routine data collected by the public health authorities in Germany to gather detailed information on variant behavior at an early stage.

The rapid identification of new variants or subvariants of SARS-CoV-2 is important for surveillance, in order to closely monitor the occurrence of infection and to enable initiating protective countermeasures. Timely investigations of public health data connected to confirmed circulating SARS-CoV-2 variants, including reported symptoms, enables the assessment of the current situation and the chance to react quickly to manage the pandemic.

## Figures and Tables

**Table 1:** Epidemiological characteristics and analyses on information from recorded BA.2 and BA.5 cases.

Characteristics		BA.2			BA.5		*p* Value ^1^
		*n*	[%]		*n*	[%]	
Sex	Female	126	51.2		96	60.4	0.135
	Male	118	48.0		61	38.4	
	Unknown	2	0.8		2	1.3	
Age groups	0–19	25	10.2		13	8.2	0.759
	20–39	76	30.9		48	30.2	
	40–59	94	38.2		70	44.0	
	60–79	43	17.5		24	15.1	
	80+	7	2.8		3	1.9	
	Unknown	1	0.4		1	0.6	
Symptoms	Yes	124	50.1		69	43.4	0.186
	No	122	49.9		90	56.6	
Vaccination ^2^	Yes	75	57.7		60	49.2	0.124
	No	42	32.3		54	44.3	
	Unknown	13	10		8	6.6	

^1^*p* values are shown from Pearson’s chi square statistics. ^2^ No records from 174 cases.

**Table 2:** Differences between reported symptoms from symptomatic BA.2 and BA.5 cases (CI, confidence interval (CI 95% [lower–upper]); Positive, positively reported; n.r., not reported.).

Symptom	Status	BA.2			BA.5			*p*-Value ^1^
		*n*	%	CI 95 [%]	*n*	%	CI 95 [%]	
Breathing difficulties/dyspnoea	Positive	18	14.5	[9.2–21.5]	4	5.8	[2.0–13.2]	0.097
	n.r.	106	85.5	[78.5–90.8]	65	94.2	[86.8–98.0]	
Chills or sweating	Positive	22	17.7	[11.8–25.2]	3	4.3	[1.2–11.1]	**0.012**
	n.r.	102	82.3	[74.8–88.2]	66	95.7	[88.9–98.8]	
Cough	Positive	105	84.7	[77.6–90.2]	52	75.4	[64.3–84.3]	0.125
	n.r.	19	15.3	[9.8–22.4]	17	24.6	[15.7–35.7]	
Diarrhea	Positive	12	9.7	[5.4–15.8]	9	13	[6.7–22.5]	0.631
	n.r.	112	90.3	[84.2–94.6]	60	87	[77.5–93.3]	
Fever	Positive	43	34.7	[26.7–43.3]	34	49.3	[37.7–60.9]	0.065
	n.r.	81	65.3	[56.7–73.3]	35	50.7	[39.1–62.3]	
Freeze	Positive	29	23.4	[16.6–31.4]	6	8.7	[3.7–17.0]	**0.011**
	n.r.	95	76.6	[68.6–83.4]	63	91.3	[83.0–96.3]	
Headache	Positive	68	54.8	[46.1–63.4]	37	53.6	[41.9–65.0]	0.881
	n.r.	56	45.2	[36.6–53.9]	32	46.4	[35.0–58.1]	
Increased heart rate/tachycardia	Positive	12	9.7	[5.4–15.8]	3	4.3	[1.2–11.1]	0.264
	n.r.	112	90.3	[84.2–94.6]	66	95.7	[88.9–98.8]	
Loss of smell	Positive	15	12.1	[7.2–18.7]	15	21.7	[13.3–32.5]	0.097
	n.r.	109	87.9	[81.3–92.8]	54	78.3	[67.5–86.7]	
Loss of taste	Positive	25	20.2	[13.8–27.9]	17	24.6	[15.7–35.7]	0.585
	n.r.	99	79.8	[72.1–86.2]	52	75.4	[64.3–84.3]	
Muscle or body aches	Positive	40	32.3	[24.5–40.8]	28	40.6	[29.6–52.4]	0.273
	n.r.	84	67.7	[59.2–75.5]	41	59.4	[47.6–70.4]	
Nausea	Positive	8	6.5	[3.1–11.8]	6	8.7	[3.7–17.0]	0.774
	n.r.	116	93.5	[88.2–96.9]	63	91.3	[83.0–96.3]	
Other symptoms ^2^	Positive	39	31.5	[23.8–40.0]	40	58.0	[46.2–69.1]	**<0.001**
	n.r.	85	68.5	[60.0–76.2]	29	42.0	[30.9–53.8]	
Pneumonia	Positive	2	1.6	[0.3–5.1]	0	0	[–]	0.289
	n.r.	122	98.4	[94.9–99.7]	69	100	[–]	
Rapid breathing/tachypnea	Positive	2	1.6	[0.3–5.1]	2	2.9	[0.6–9.0]	0.618
	n.r.	122	98.4	[94.9–99.7]	67	97.1	[91.0–99.4]	
Runny nose	Positive	110	88.7	[82.3–93.4]	52	75.4	[64.3–84.3]	**0.023**
	n.r.	14	11.3	[6.6–17.7]	17	24.6	[15.7–35.7]	
Severe feeling of sickness	Positive	40	32.3	[24.5–40.8]	30	43.5	[32.2–55.2]	0.159
	n.r.	84	67.7	[59.2–75.5]	39	56.5	[44.8–67.8]	
Sore throat/pharyngitis	Positive	77	62.1	[53.4–70.3]	37	53.6	[41.9–65.0]	0.538
	n.r.	47	37.9	[29.7–46.6]	32	46.4	[35.0–58.1]	

^1^ In bold: significant *p* values are shown from Pearson’s chi square statistics. ^2^ ‘Other symptoms’ are explained in the text.

**Table 3:** Non-parametric correlation between different symptoms of both BA.2 and BA.5 positive cases. Spearman-Rho correlation coefficients > 0.3. are given in bold.

	Symptoms												
	Chills or Sweating	Cough	Diarrhea	Breathing Difficulties/Dyspnea	Fever	Headache	Muscle or Body Aches	Rapid Breathing/Tachypnea	Runny Nose	Sore Throat/Pharyngitis	Loss of Taste	Loss of Smell	Freeze
Chills or sweating	1.000												
Cough	0.026	1.000											
Diarrhea	0.063	0.082	1.000										
Breathing difficulties/dyspnea	0.299 ^2^	0.046	0.136	1.000									
Fever	0.095	0.064	0.191 ^2^	0.107	1.000								
Headache	0.198 ^2^	0.096	0.019	0.230 ^2^	0.278 ^2^	1.000							
Muscle or body aches	0.232 ^2^	0.047	0.125	0.179 ^1^	0.174 ^1^	**0.414 ^2^**	1.000						
Rapid breathing/tachypnea	0.161 ^1^	0.07	0.066	0.291 ^2^	0.179 ^1^	0.133	0.121	1.000					
Runny nose	0.169 ^1^	**0.334 ^2^**	0.108	0.113	−0.105	0.053	−0.002	0.064	1.000				
Sore throat/pharyngitis	0.07	0.169 ^1^	0.122	0.1	0.205 ^2^	0.190 ^2^	0.217 ^2^	−0.027	0.181 ^1^	1.000			
Loss of taste	0.096	0.059	0.219 ^2^	0.127	0.109	0.079	0.242 ^2^	0.188 ^2^	0.094	0.107	1.000		
Loss of smell	0.133	0.022	0.263 ^2^	0.251 ^2^	0.147 ^1^	0.163 ^1^	0.192 ^2^	0.239 ^2^	0.149 ^1^	0.124	**0.571** **^2^**	1.000	
Freeze	**0.579** **^2^**	0.122	0.181 ^1^	0.254 ^2^	0.111	0.269 ^2^	**0.328** **^2^**	0.12	0.206 ^2^	0.009	0.045	0.021	1.000

^1^ Significant correlations *p* < 0.05. ^2^ Significant correlations *p* < 0.01.

## Data Availability

Not applicable.
